# Methylation array profiling of adult brain tumours: diagnostic outcomes in a large, single centre

**DOI:** 10.1186/s40478-019-0668-8

**Published:** 2019-02-20

**Authors:** Zane Jaunmuktane, David Capper, David T. W. Jones, Daniel Schrimpf, Martin Sill, Monika Dutt, Nirosha Suraweera, Stefan M. Pfister, Andreas von Deimling, Sebastian Brandner

**Affiliations:** 1 Division of Neuropathology, National Hospital for Neurology and Neurosurgery, University College London Hospitals NHS Foundation Trust, Queen Square, London, WC1N 3BG UK; 2 Department of Clinical and Movement Neurosciences, UCL Queen Square Institute of Neurology, Queen Square, London, WC1N 3BG UK; 3 Department of Neuropathology, Charité Universitätsmedizin Berlin, Corporate Member of Freie Universität Berlin, Humboldt-Universität zu Berlin and Berlin Institute of Health, Berlin, Germany; 4 German Cancer Consortium (DKTK), Partner Site Berlin, German Cancer Research Center (DKFZ), Heidelberg, Germany; 5 Hopp Children’s Cancer Center Heidelberg (KiTZ), Heidelberg, Germany; 6 Pediatric Glioma Research Group, German Cancer Research Center (DKFZ), Heidelberg, Germany; 7 Department of Neuropathology, University Hospital Heidelberg, Heidelberg, Germany; 8 Clinical Cooperation Unit Neuropathology, German Cancer Consortium (DKTK), German Cancer Research Center (DKFZ), Heidelberg, Germany; 9 Division of Pediatric Neurooncology, German Cancer Research Center (DKFZ), Heidelberg, Germany; 10 Department of Pediatric Oncology, Hematology, Immunology and Pulmonology, Heidelberg University Hospital, Heidelberg, Germany; 11 Department of Neurodegenerative Disease, UCL Queen Square Institute of Neurology, Queen Square, London, WC1N 3BG UK

**Keywords:** IDH1, IDH2, BRAF, Histone mutation, H3 K27M, Methylation array, Illumina array, DKFZ classifier, Methylation classifier, Molecular diagnostics, Glioma, Ependymoma, Brain tumour classification, Adult brain tumours

## Abstract

The introduction of the classification of brain tumours based on their DNA methylation profile has significantly changed the diagnostic approach for cases with ambiguous histology, non-informative or contradictory molecular profiles or for entities where methylation profiling provides useful information for patient risk stratification, for example in medulloblastoma and ependymoma. We present our experience that combines a conventional molecular diagnostic approach with the complementary use of a DNA methylation-based classification tool, for adult brain tumours originating from local as well as national referrals. We report the frequency of IDH mutations in a large cohort of nearly 1550 patients, EGFR amplifications in almost 1900 IDH-wildtype glioblastomas, and histone mutations in 70 adult gliomas. We demonstrate how additional methylation-based classification has changed and improved our diagnostic approach. Of the 325 cases referred for methylome testing, 179 (56%) had a calibrated score of 0.84 and higher and were included in the evaluation. In these 179 samples, the diagnosis was changed in 45 (25%), refined in 86 (48%) and confirmed in 44 cases (25%). In addition, the methylation arrays contain copy number information that usefully complements the methylation profile. For example, EGFR amplification which is 95% concordant with our Real-Time PCR-based copy number assays. We propose here a diagnostic algorithm that integrates histology, conventional molecular tests and methylation arrays.

## Introduction

The diagnosis of brain tumours is achieved by combining morphological features, immunohistochemical (IHC) detection of lineage-related markers, and more recently by the detection of genetic biomarkers, for example mutations in the isocitrate dehydrogenase genes 1 and 2 (*IDH1* and *IDH2*) [1, 13], *BRAF* [35], or histone genes [16]. The development of mutation-specific antibodies to the most common IDH1 mutation R132H [5], BRAF V600E [3] or Histone H3 K27 M [6] has facilitated the introduction of these tests into routine neuropathology diagnostics and their use is our first diagnostic step. To refine the diagnostic accuracy, we use Sanger sequencing, for example to detect rarer mutations in the *IDH1*, or *IDH2* genes [26], histones [16], or to detect mutations in the *TERT* promoter either to support glioma diagnostics in the context of other mutations [10] or to prognosticate meningioma recurrence risk [33]. Yet, a significant number of CNS tumours still lack distinctive, and diagnostically informative mutations that can be readily implemented into routine diagnostic practice, or such tests (e.g. gene fusion tests covering multiple breakpoints) may be resource-intensive to set up, validate and to test routinely. Therefore, neuropathologists may be tempted to revert to the traditional approach of tumour typing and grading, which is fraught by considerable intra-, and inter-observer variability, and by a lack of robust clinical-pathological correlation. For example, it is well established that grading based on histological features such as mitotic counts, cellularity, pleomorphism, vascular abnormalities and necrosis do not correlate well with the clinical outcome in ependymomas [24] or in diffuse gliomas [38]. The prognostication of intrinsic brain tumours based mainly or exclusively on morphology can be misleading, as for example shown in a large-scale study on IDH-wildtype low-grade astrocytomas, where a small proportion was confirmed to be of low-grade, whilst a much larger proportion corresponded molecularly to high-grade gliomas [29]. The ambiguity of traditional histopathological criteria to inform clinical oncologists on patient management, and the patients of the prognosis, called for a radically new approach for tumour diagnostics, leading to the development of a comprehensive CNS tumour reference cohort based on genome-wide DNA methylation profiles [2, 4].

Methylation profiles of tumours result from a combination of somatically acquired DNA methylation changes and the cell of origin [11]. These profiles are highly robust and reproducible in clinical pathology settings [15] and have been widely used to subclassify CNS tumours, for example ependymomas [25], meningiomas [34], medulloblastomas [15], nerve sheath tumours [31], primitive neuroectodermal tumours [40] or other tumour types such as “small blue round cell tumours” [17]. A brain tumour methylation classifier has been developed at the German Cancer Research Center (DKFZ) and Heidelberg University in Heidelberg, Germany (henceforth in short “Classifier”), to identify distinct DNA methylation classes of CNS tumours. Currently, the Classifier comprises 82 CNS tumour methylation classes and nine control tissue methylation classes [2]. The Classifier has been made available through a free online tool (www.molecularneuropathology.org). We have used this classification tool [2, 4] in clinical practice to stratify into clinically relevant risk groups of histologically defined CNS (and related) tumour entities, and as an aid to establishing a diagnosis in histologically uncertain cases, for example when morphology, location and demographics were highly unusual, the histology non-specific, or where molecular tests were contradictory, ambiguous or non-informative. We have analysed more than 500 tumours using Illumina 450 K or 850 K EPIC methylation arrays, followed by algorithmic classification with the Classifier. Here we present the implementation of this platform in a neuropathology department within a major academic health science centre, and our experience using the Classifier in routine clinical diagnostic practice.

## Material and methods

### The rationale for methylation profiling and tumour selection

Methylation profiling was set up in the Division of Neuropathology, the National Hospital for Neurology and Neurosurgery (NHNN) at University College London Hospitals NHS Foundation Trust, a large clinical centre for neurological disorders of adults. Tumours included in this study were analysed with methylation arrays between February 2015 and November 2018.

Samples included in this study originated from our own hospital (NHNN) or were referred to us for a second opinion or conventional and advanced molecular profiling. All tumours underwent routine histopathological assessment, including immunohistochemical and molecular examination in our centre or by referring pathologists, and then were processed for methylation arrays. The entry criteria for methylation profiling of diagnostic samples were not strictly predefined but were guided by a routine diagnostic decision-making process. A proportion of cases was profiled in the context of research studies or clinical trials, and these were not included in this study.

The cases submitted for the methylation arrays were categorised into six groups with the following characteristics: (i) unusual combination of morphology, location and demographics, (ii) contradictory, ambiguous or non-informative molecular tests, (iii) confirmation of unusual histological and molecular results, (iv) small biopsy or non-representative sample, (v) indistinct or non-specific histological appearance, and (vi) cases with characteristic histology requiring risk stratification, such as medulloblastoma [41] or ependymoma [24].

In order to gain experience with the implementation of the technology in our laboratory, and to correlate clinical, pathological and molecular features with the results of the Classifier; a proportion of tumours with clearly defined (molecular) biomarker profile was used to set up and validate the procedures. During our setup phase, the Classifier was also used for re-classification of tumours previously diagnosed as oligoastrocytoma, which has been discontinued as a distinct entity [30, 32, 42], or tumours with the histological phenotype of adult primitive neuroectodermal tumour (PNET) which now resolve into multiple different entities [40].

### Specimen preparation and quality control

All tissues used for methylation studies were fixed in formalin for at least 4 h, and larger samples were dissected and fixed overnight, followed by processing through graded alcohols and xylene, to paraffin according to standard practice in an ISO15189 accredited laboratory. Tissue embedding and sectioning were according to standard histopathology procedures.

### Selection of tumour area

Sections of the formalin fixed paraffin embedded (FFPE) samples selected for methylation array analysis were mounted on glass slides (by default 10 μm thickness, 8 consecutive slides). On a consecutive H&E stained section (3–4 μm), a suitable tumour area was identified by a neuropathologist (SB or ZJ), to maximise inclusion of viable tumour-containing tissue. Tumour content of at least 80% was selected where possible and non-neoplastic tissue, blood or excessive areas of necrosis were excluded. However, on some occasions where the specimen contained an overall lower tumour density (e.g. infiltration zone) a methylation array analysis was nevertheless attempted, acknowledging a potential risk of an inconclusive Classifier result.

### DNA extraction and quantification

Slides with mounted tissue were dewaxed (3 washes in xylene and 2 washes with industrial methylated spirit) and air-dried. Tissue selected for the analysis was scraped off and collected in lysis buffer and DNA was extracted with the Maxwell 16 Lev FFPE DNA Purification Kit on a Maxwell 16 extractor [19]. The DNA extraction procedure was carried out according to manual #TM349 for DNA extraction (Promega). DNA was then quantified and A_260_/A_280_ ratios were determined on a Nanodrop 8000 Spectrophotometer (ThermoFisher). An A_260_/A_280_ ratio of ~ 1.8 was considered to represent sufficient purity to proceed with the methylation study. However, rarely we also process samples with a lower A_260_/A_280_ ratio if there is a clinical necessity and no additional material available to repeat extraction or purification.

In our practice, tissue size and resulting DNA amount was rarely the limiting factor. Even a single core of a small stereotaxic biopsy, extracted from 8 consecutive sections of 10 μm thickness yielded well above the recommended minimum of 250 ng. A single core of approximately 4 mm^2^ (calculated tissue volume 0.34 mm ^3^) has yielded 600 ng of high-quality DNA, and slightly larger cores of 10–12 mm^2^ (calculated tissue volume 0.8–0.9 mm^3^) have yielded 1400–1800 ng DNA, i.e. well above 250 ng. All these examples were processed and returned a result with a calibrated score of 0.99. In our practice we aim at a DNA input of 500 ng, and in our experience a limiting factor is more often the tissue (and resulting DNA) quality, or tumour content, rather than sample size.

### FFPE tissue quality control (QC) assay

Real-time PCR (RT-PCR) assays were run with technical triplicates using DNA isolated from FFPE samples and a QC standard, using primers supplied in the Illumina Infinium HD FFPE QC Kit (Infinium HD FFPE QC Assay Protocol, Illumina). The quality cycle threshold (QCT) value was calculated by subtracting the average Cq of Illumina QC standard from the average Cq value determined for each FFPE sample. Illumina recommends that a QCT value ≤5 be utilized for optimal assay performance.

### Bisulphite conversion of DNA

Based on the DNA quantification steps as determined previously, we aim at an input of 250 ng as a minimum, and ideally at 500 ng DNA from each sample for bisulphite conversion. The EZ DNA Methylation™ Kit (Zymo D5024) was used for DNA conversion. All steps were performed according to the manufacturer’s guidelines.

### Copy number assays and sequencing

DNA for copy number assays or direct sequencing was extracted from FFPE tumour tissue using Maxwell 16 FFPE LEV DNA purification kit (Promega). Tumour area was confirmed on an H&E-stained slide and tissue was microdissected from consecutive 10 μm FFPE sections. Primer design was as follows: IDH1-F ACCAAATGGCACCATACGA; IDH1-R TGCTTAATGGGTGTAGATACCAAA; IDH2-F CCAATGGAACTATCCGGAAC; IDH2-R TGTGGCCTTGTACTGCAGAG, BRAF 600-f TCATAATGCTTGCTCTGATAGGA; C600-r GGCCAAAAATTTAATCAGTGGA, TERT-f AGTGGATTCGCGGGCACAGA, TERT-R; Histone H3F3-F CATGGCTCGTACAAAGCAGA, H3F3-R CAAGAGAGACTTTGTCCCATTTTT. For all copy number assays we used the Comparative CT (threshold cycle) multiplex PCR (in same tube) method (ΔΔCT) [36]. The following probes were used for target and reference genes, respectively: 1p36.12b (assay ID Hs06545466_cn; RnaseP 4401631), 1p13.3a (assay ID Hs01847890_cn; RnaseP 4401631); 19q13.2b (assay ID Hs00954642_cn; RnaseP 440163); 19q13.42c (assay ID Hs00831101_cn; RnaseP 440163); 10q23.31a (assay ID Hs05203872_cn; RnaseP 440163); 7p11.2c (assay ID Hs01381289_cn; TERT 4401633). Calibrators were commercial human genomic DNA (gDNA) at a concentration of 10 μg/μl, (Human Genomic DNA (Male), Promega, G147a) and mixed DNA (mDNA), which contains 1:3 dilution of the gDNA. Copy numbers were determined with the CopyCaller® Software v2.1 (Applied Biosystems).

### Immunohistochemistry

All IHC stainings were carried out on automated immunostainers (Roche Ventana Discovery or LEICA BondMax) following manufacturer’s guidelines. The IDH1 R132H, BRAF V600E, H3 K27M and ATRX antibodies were used as published [3, 6, 30].

### Performing Infinium FFPE restoration

Degraded FFPE DNA was restored into an amplifiable condition with the Infinium HD FFPE DNA Restore Kit (24 samples, WG-321-1002) according to the manufacturer’s instructions.

### Array processing

The 450 k or EPIC (850 k) methylation array was used to obtain genome-wide DNA methylation profiles for FFPE tumour samples, according to the manufacturer’s instructions (Illumina). DNA methylation data were generated at the UCL genomics facility at UCL Institute of Child Health. On-chip quality metrics of all samples were carefully controlled. Data (idat files) were transferred to the Division of Neuropathology and uploaded to the Classifier (www.molecularneuropathology.org). Following the upload, the classification result was returned automatically as reported [2].

## Results and discussion

### Definition of outcomes and calibrated score

**Fig. 1:**
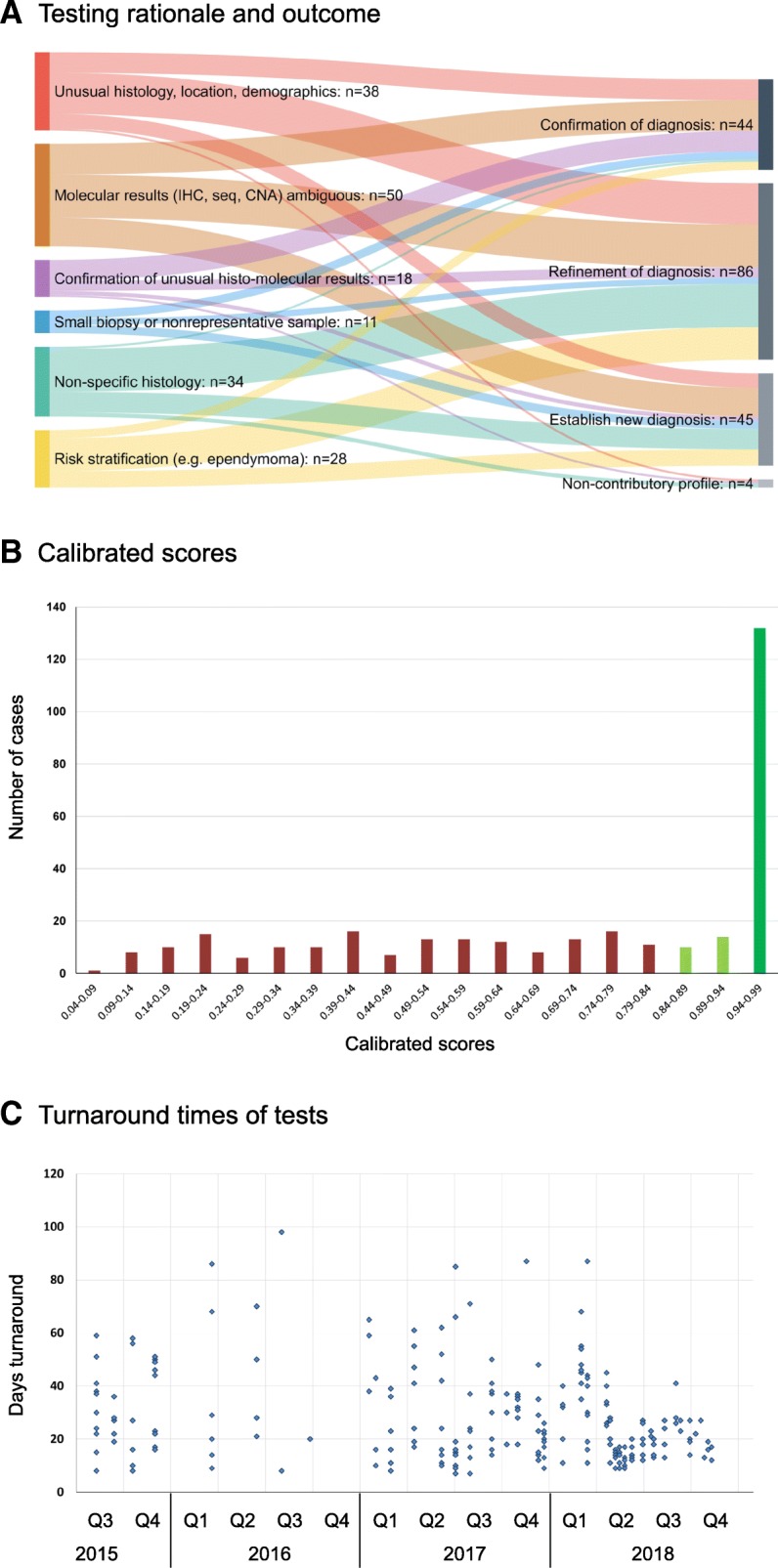
**a**, the association of methylation array testing rationale (left) with the outcome of the methylome-based classification (right). 179 cases with a calibrated score of 0.84 and higher were included in this graph. **b**, distribution of calibrated scores in 325 diagnostic samples examined (excluding research samples) demonstrating that for > 55% (179/325) of the predictions the classifier has had high confidence with estimated class probabilities of 94 > 99%. **c**, Scattergram of turnaround times (TAT) of tests between 2015 and 2018. Whilst the TAT in the first 2 years of the setup phase often comprised 50% of latencies over 30 days, these long TAT have been progressively reduced in 2018. In quarters 2–4 of 2018, the majority of the cases was completed within 4 weeks. For the first four months in 2015 we did not record the dates of requesting tests, therefore no TAT are shown between February and June 2015

For best comparison with other datasets, we aligned the definitions closely to the initial publication of the classification tool [2]. The outcomes were classified according to the impact on the original pathological diagnosis: original pathology confirmed (outcome 1), refined (outcome 2) or a new diagnosis established (outcome 3). Alternatively, the Classifier result was considered misleading (outcome 4) or inconclusive (outcome 5) (Fig. 1a and Table 1). The frequency of outcome 4 or 5 depends on the threshold of the calibrated score. We included in our analysis only results with a calibrated score of 0.84 and above as recommend in [4]. Classifier results with a calibrated score below 0.84 can still yield informative results [4], in particular when taking into account copy number profiles (such as 7p gain; 10q loss in IDH-wildtype glioblastoma, 1p/19q codeletion in IDH-mutant oligodendroglioma, or copy number variation and *CDKN2A/B* deletions in IDH-mutant astrocytomas). Calibrated scores are class probability estimates that measure confidence in the prediction. If the score calibrated is working perfectly, among all tumours of a “Class X” with a score of 90%, there will be 90% “Class X” tumours. A low score indicates that the classifier is uncertain in its prediction and thus these predictions are often false. Otherwise, if most of the low score predictions were true, the probability estimation (or score calibrated) would not work correctly.

**Table 1:** Number of cases referred for methylation array analysis and the proportion of cases with a calibrated score of 0.84 and higher, and the proportion of cases in which the diagnosis was changed, refined, or confirmed or where the test yielded non-contributory results

	Number of cases	Proportion of all cases	Proportion of cases with CS > 0.84
Cases referred for testing	325	100%	56%
Tumours with a calibrated score > 0.84	179	56%	100%
Establishing new diagnosis	45	14%	25%
Refinement of diagnosis	86	26%	48%
Confirmation of diagnosis	44	14%	25%
Non-contributory	4	1%	2%

*CS* Calibrated score

**Confirmation of diagnosis (outcome 1)**: this category includes cases in which the Classifier confirmed the integrated diagnosis, such as IDH-mutant and 1p/19q co-deleted oligodendroglioma, IDH-mutant glioblastoma, subependymoma, H3 K27M-mutant diffuse midline glioma, and similar. This category also comprises tumour entities where histology or location of a tumour is unusual.

**Refinement of diagnosis (outcome 2)**: this category includes cases in which the Classifier confirms the histological or integrated diagnosis, and determines a more specific molecular subtype, for example in IDH-wildtype glioblastoma, ependymoma or medulloblastoma. It also includes cases where the diagnostic accuracy is improved, e.g. confirmation of an IDH-mutant oligodendroglioma with previously tested ambiguous 1p/19q result or sub-classification of tumours with non-specific low-grade morphology, such as IDH-wildtype low-grade glioneuronal tumours.

**Establishing a new diagnosis (outcome 3)**: this outcome was assigned to indicate a change of the original diagnosis (usually unexpected, for example, the change from the histological diagnosis of an ependymoma to the methylation class of a pleomorphic xanthoastrocytoma, PXA). The main reasons for this outcome were (i) morphologically inconclusive specimens (e.g. with a diagnosis of low-grade or high-grade glioma, with no specific molecular alterations detectable by conventional molecular methods), for which a conclusive methylation class could be established; (ii) incorrect histological interpretation, where relevant tests were not considered as a consequence (e.g. solitary fibrous tumour/haemangiopericytoma misdiagnosed as meningioma); (iii) a distinct histological pattern does not correspond to a specific methylation class (e.g. a tumour with the pattern of astroblastoma often but not always belongs to the HGNET_MN1 methylation class); (iv) all tumours with a newly defined methylation class that do not correspond to an existing WHO entity (e.g. “primitive neuroectodermal tumour” (PNET) resolving into multiple newly defined entities).

**Misleading profile (outcome 4)**: in our experience, there are two scenarios in which misleading results can occur. A low calibrated score can result in a methylation class that is inconsistent with previously tested molecular markers and histology. For example, the methylation class IDH-wildtype glioma in a previously confirmed IDH-mutant glioma. A high calibrated score of > 0.84 rarely generates misleading results. In our 325 diagnostic samples, 179 (56%) had a calibrated score of > 0.84 (Fig. 1b) and none of these showed a misleading profile.

**Inconclusive or non-contributory profile (outcome 5)**: was assigned to cases that showed an obvious discrepancy between the input material and the methylation class (such as normal control tissue in cases where analysed material was of a tumour) or in cases where the methylation profiling did not provide any additional diagnostic information. In cases with calibrated score > 0.84 we encountered four cases with a non-contributory profile.

### Integration of methylation classification into the diagnostic process

Below we outline our diagnostic workflow and decision-making process for adult CNS tumours, incorporating the use of methylation arrays and the Classifier. Our department receives diagnostic samples through three pathways: (i) the local hospital (NHNN) refers tissue for the complete diagnostic workup (tissue diagnosis, molecular diagnostics (pathway 1)); (ii) two geographically separate clinical centres refer formalin-fixed tissue for complete workup as above (pathway 2); and (iii) direct referral of externally diagnosed tumours for advanced molecular workup, often with a specific request to perform methylation array analysis (pathway 3).

### Turnaround times

An important consideration for clinical utility is the turnaround time of tests. The turnaround times (TAT) of methylation arrays are partly dictated by the necessity to form batches (12 arrays on the 450 k chips, and 8 arrays on the 850 k chips), the time it takes to process chips, and how often arrays are processed in a genomics facility. The TAT also depends on the accrual rate of samples. Figure 1c shows a graphical representation of the TAT (the time required from ordering the test in the laboratory to receiving the data files for the upload on the webpage). Figure 1c shows our institutional performance between 2015 and 2018, demonstrating a relatively significant variation in sample throughput (which is directly proportional to the number of requested tests) and the TAT. Over time, practice and workflow optimisation has reduced the proportion of samples with TAT exceeding 30 days.

### Cost implications

Using list prices for arrays and conversion kits and facility fees, processing of one sample incurs a cost of approximately £380 as of November 2018, without applying discounts that are currently available to our institution. These are itemised as follows: Microarray WG-317-1003 £267 per sample for orders of 96 samples; FFPE restore Kit WG-321-1002 £64 per sample (kit for 24 samples), salary cost for sample registration, DNA extraction, bisulphite conversion, quality control, data upload and results download (8 h @ £50/h = £400, or £50 per sample). This does not include time for medical staff to report cases, taking approximately 30–45 min per case. We consider methylation arrays as a cost-effective and tissue-saving approach for diagnostically challenging cases. A single IHC section costs approximately £18 (full economic costing) and methylation arrays are likely to yield significantly more information and thus better value for money than large panels of immunostains. In our practice, where methylation arrays are readily available and embedded in the diagnostic pathway we usually do not perform more than 10 immunostainings on intrinsic tumours, as additional stains are unlikely to add meaningful information. An even lower threshold (as few as 3–5 immunostains) is applied to small, precious samples such as stereotaxic biopsies, and targeted sequencing and methylation arrays are considered early in the diagnostic workup. However, for the routine molecular diagnostics where such limitations do not apply, methylation arrays are not yet the first choice: the consumable cost for Sanger sequencing and copy number assays is a fraction of those for methylation arrays, and the turnaround times are significantly shorter, making it impractical and unaffordable to implement methylation arrays for the diagnosis of all brain tumours.

**Local tissue referrals (pathways 1 and 2):** all brain tumours undergo routine histological and immunohistochemical examination and are reported as part of the standard diagnostic process in our department. Low- and high-grade gliomas and poorly differentiated supratentorial intrinsic tumours with PNET morphology undergo immunostaining for IDH1 R132H and ATRX. IDH-mutant tumours with ATRX loss are diagnosed as IDH-mutant astrocytomas or glioblastomas (GBM), and for prognostication, these are tested for *CDKN2A/B* homozygous deletion [38]. IDH-mutant tumours with retained ATRX undergo further testing for 1p/19q and *TERT* promoter mutations. Midline gliomas are routinely tested with immunostaining for H3 K27M. IDH1 R132H negative gliomas undergo a targeted sequencing for known mutations in the *IDH1/2*, histone H3.3 (*H3F3A*), *BRAF* genes and the *TERT* promoter, and copy number assays (1p/19q, *CDKN2A/B*, 7p (*EGFR*), and 10q (*PTEN* locus)). This identifies the remaining IDH- or histone-mutant gliomas and IDH-wildtype, *TERT*-mutant, and/or *EGFR* amplified GBM. IDH- and histone-wildtype gliomas with ATRX loss also undergo *BRAF* fusion testing to identify possible anaplastic astrocytoma with piloid features [28].

Tumours with unusual location or non-specific glial or glioneuronal morphology and non-informative conventional molecular test results are then considered for methylation arrays. In this study, the threshold to use the Classifier was lower for young adults, although we did not define a specific age cut-off and instead made a case-based decision in consultation with the clinical teams. The Classifier was also used for confirmation of rare tumour entities which do not have any of the above-mentioned gene mutations. The methylation class was reported as part of the integrated diagnosis, e.g. “histology: low-grade glioma, IDH-wildtype; methylation class: glioblastoma, IDH-wildtype RTK II”; or “histology: high-grade glioma; methylation class: pleomorphic xanthoastrocytoma; BRAF V600E mutant”.

All supra- and infratentorial ependymomas undergo methylation studies. We encounter a relatively small number of supratentorial ependymomas in our adult practice (in this cohort, from pathways 1 and 2 *n* = 7), which in our view justifies initial risk stratification by methylation profiling [12] instead of nucleic acid-based tests for multiple potential gene fusion transcripts. Tumours with characteristic subependymoma morphology irrespective of location and all spinal ependymomas are not tested further unless there is a specific clinical request, an unusual clinical presentation, or histology.

Infratentorial tumours with features of embryonal tumours (e.g. medulloblastoma), similar to ependymomas, typically are processed for methylation array for risk stratification purposes, although the prognostic significance of medulloblastoma subclasses in adults is currently not firmly established. Since April 2018, all adult medulloblastomas in the UK are referred to the National Medulloblastoma Reference Centre through Great Ormond Street Hospitals [37].

Pilocytic astrocytomas are routinely tested for *BRAF* V600 and the three most common *KIAA1549:BRAF* fusion mutations (16–9, 16–11, 15–9). Tumours with a confirmed mutation are not further investigated. Tumours with pilocytic astrocytoma or other low-grade glial or glioneuronal morphology with no *BRAF* V600 mutation, absence of the three tested fusions, and in particular, those with loss of ATRX protein expression routinely undergo methylation array testing.

**Referrals of previously diagnosed tumours (pathway 3)** are (i) specifically referred for Illumina array analysis, or received for (ii) targeted diagnostics with conventional molecular tests as above, or (iii) second opinion, often with extensive previous workup, including molecular testing. Cases which remain inconclusive after the molecular studies are then processed for methylation arrays and an integrated diagnosis is returned to the referring pathologists.

### Diagnostic outcomes from molecular assays in our practice

#### IDH-mutant gliomas

The most common reason for further workup with methylation array is a confirmed *IDH* mutation in combination with an inconclusive ATRX, *TERT* promoter and 1p/19q status; for example IDH-mutant gliomas with retained ATRX expression and ambiguous 1p/19q status; or rare diffuse gliomas with ATRX loss in which the *IDH*- or histone H3 mutation cannot be established by IHC or sequencing.

**Fig. 2:**
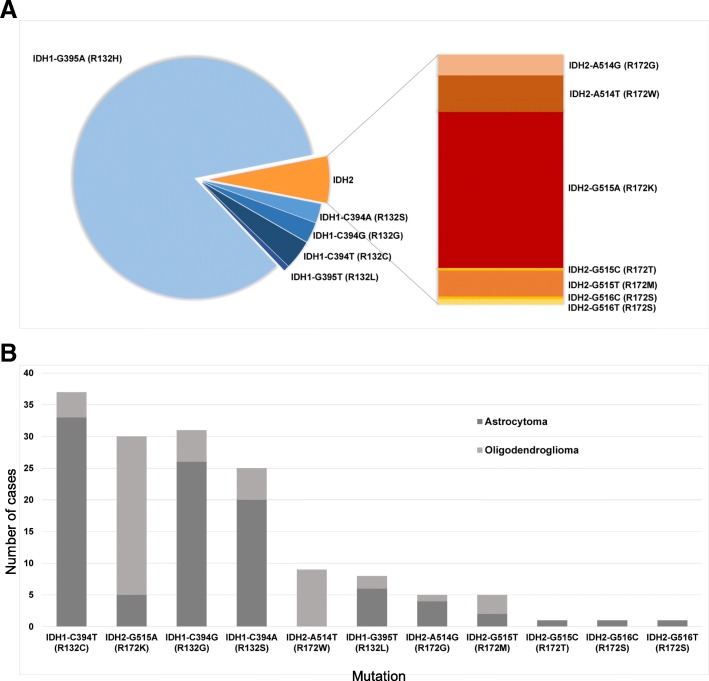
**a**, the frequency of IDH mutations in our cohort (*n* = 1546). Blue, *IDH1* mutations; orange and red, *IDH2* mutations. The *IDH1/IDH2* frequency in our cohort is slightly skewed toward rarer mutations, due to a proportion of referrals received specifically for sequencing studies. **b**, the frequency of *IDH1* and *IDH2* mutations and associated tumour types (*n* = 441 astrocytomas (of which 339 have *IDH1*-G395A); *n* = 363 oligodendrogliomas (of which 303 have *IDH1*-G395A)). Dark grey, astrocytomas; light grey, oligodendrogliomas. The mutations are sorted in descending order by overall frequency, excluding the most common *IDH1*-G395A mutation. The graph confirms the established association of certain mutations, in particular in the *IDH2* gene, with oligodendroglial or astrocytic tumours

**Fig. 3:**
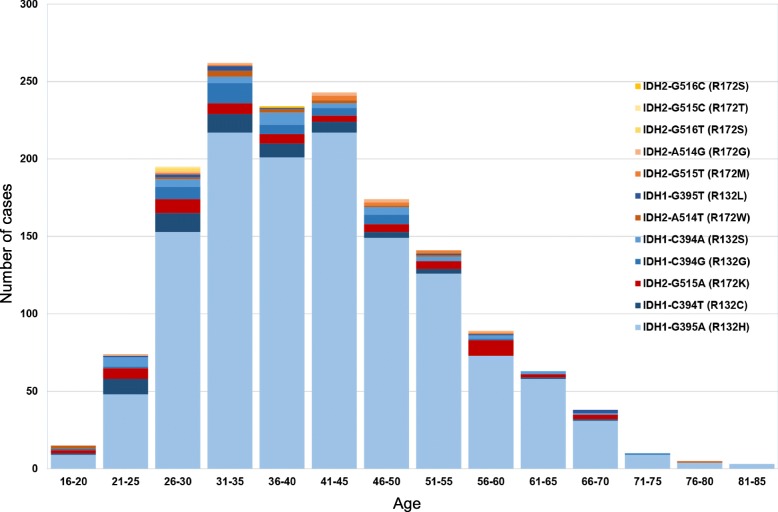
Age distribution of IDH mutations in our cohort (*n* = 1546) demonstrates that 15.7% of all *IDH1/IDH2* mutations occur in patients 55 years and older, justifying routine testing for these mutations in this age cohort

In order to establish if an upper age limit can be justified for the testing of *IDH1/IDH2* mutations beyond the use of immunostaining with the IDH1 R132H mutation-specific antibody, we analysed the age distribution in 1546 tumours. The frequency distribution of the distinct *IDH1* and *IDH2* mutations is comparable to previous reports of similar scale [13] (Fig. 2). In addition to mutations described in previous series [7, 14, 26] we found rare mutations such as *IDH2-G515C* (R172T)*, IDH2-G516C* (R172S), *IDH2-G516T* (R172S), *IDH2-A514G* (R172G), and *IDH2-G515T* (R172M) in astrocytomas and oligodendrogliomas, and we found a single case with a silent mutation *IDH2-G516A* (R172R). In keeping with previous studies [13, 23] in our cohort, 243 (15.7% of the entire cohort) of IDH-mutant gliomas occurred in patients aged 55 years and older (Fig. 3), of which 31 (13%) would not have been detected by IDH1 R132H IHC alone. In our opinion, this justifies molecular testing for these rarer *IDH1/IDH2* mutations in patients over 55 years, in contrast to previous recommendations [9, 22].

As previously shown [20] and in our experience, there is a small subset of IDH-mutant, 1p/19q non-co-deleted tumours with retained ATRX protein expression. These tumours correspond to methylation class “IDH-mutant astrocytoma, high-grade”, in keeping with earlier studies [18]. Importantly, mutations in *TERT* promoter may be observed in these tumours [18].

We and most other centres use ATRX IHC as a surrogate marker for *ATRX* mutations, however, the loss of function mutations with retained protein expression are not detected [21]. As shown in our cohort and other studies [21], such mutations are relatively infrequent but provide an explanation for the discrepancies between *ATRX* sequencing and immunohistochemistry. Centres with access to affordable next-generation sequencing may consider testing such cases for the presence of a mutation.

In our cohort, we observed mutations in the *TERT* promoter in all IDH-mutant, 1p/19q co-deleted oligodendrogliomas, where testing was successful. Although the finding of *TERT* promoter mutation in IDH-mutant glioma is helpful in confirming the diagnosis of an oligodendroglial tumour, in our experience the sequencing of *TERT* promoter mutation can be technically challenging. If 1p/19q and *TERT* promoter testing remains inconclusive, sometimes occurring in referred cases that underwent different fixation protocols, we resort to further analysis with the Classifier.

#### Histone H3-mutant gliomas

**Fig. 4:**
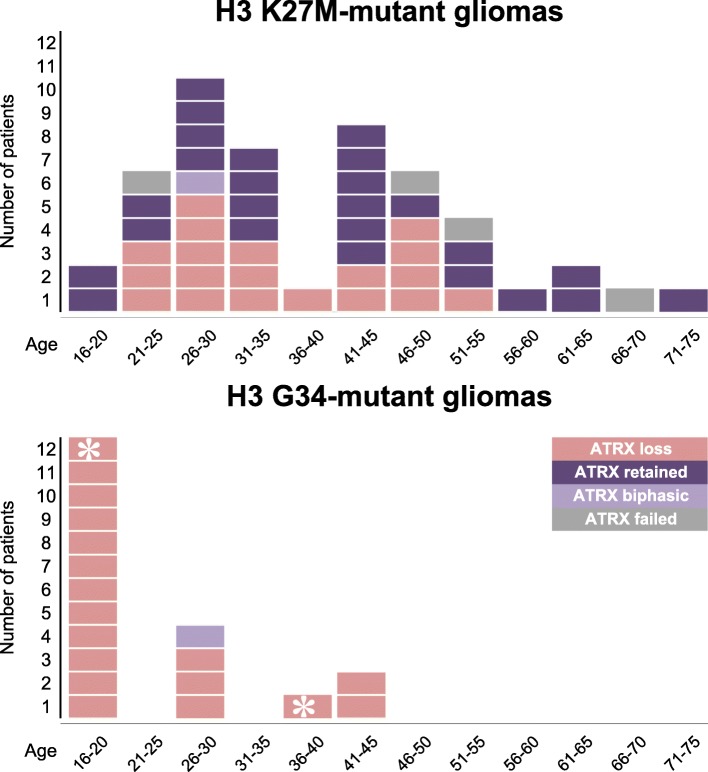
Occurrence of histone H3.3 K27M- (upper panel) and H3.3 G34-mutant gliomas in our cohort and the association with ATRX protein loss. Rarely, a biphasic pattern of ATRX expression is observed both in H3 K27M- and H3 G34R-mutant gliomas (light purple boxes). On one occasion we have identified *H3F3A* G34V mutation (asterisk in the age group 16–20 years). In another recurrent high-grade glioma, both primary and recurrent tumours were classified as H3 G34-mutant glioblastoma, although no mutations could be found in *H3F3A*, *HIST1H3B* and *HIST1H3C* genes (asterisk in the age group 36–40)

As part of the sequencing panel described above, in 2015 we introduced *H3F3A* testing in all low- and high-grade gliomas. In our practice, all H3 K27M mutant gliomas (*n* = 49) have been located in the midline, its proximity, or there was an anatomical connection to the midline. Occasionally, the proximity to the midline was *post-hoc* suggested after detection of the H3 K27M mutation. When a histone mutation is detected (by sequencing or H3 K27M IHC), we currently do not proceed to methylation array testing. Instead, midline tumours with no H3 K27M mutation, with or without ATRX loss, and no other specific findings on conventional molecular testing undergo further methylation studies. In our cohort, H3 mutant gliomas (K27M in particular) manifest also in adults over 50 years (Fig. 4). In our experience, loss of ATRX protein expression occurs in nearly all (18/19; 95%) H3 G34 mutant gliomas but only in a subset (19/45; 42%) of H3 K27M mutant gliomas. Rarely, we observed biphasic patterns of ATRX loss in H3 K27M (1 case) - and H3 G34R mutant gliomas (1 case) (Fig. 4). In another case of a recurrent high-grade glioma with ATRX loss, the methylation class was “H3 G34 mutant glioblastoma”, but we could not identify any H3 G34 mutation on the *H3F3A*, *HIST1H3B* and *HIST1H3C* genes. This raises the possibility that there is a mutation in another, as yet unknown, H3 variant-encoding histone gene.

#### Brain tumours with loss of ATRX protein expression

In our cohort, the brain tumours with loss of ATRX protein expression are IDH-mutant low- and high-grade astrocytomas, H3 K27M and G34 mutant gliomas, anaplastic pilocytic astrocytomas (methylation class ANA_PA, also termed anaplastic astrocytomas with piloid features) [28], and rarely IDH-wildtype glioblastomas, confirmed with the Classifier. Tumours with ATRX loss but no *IDH* or Histone mutation (by IHC or sequencing) generally undergo methylation array testing.

#### Newly established methylation classes representing new entities

**Fig. 5:**
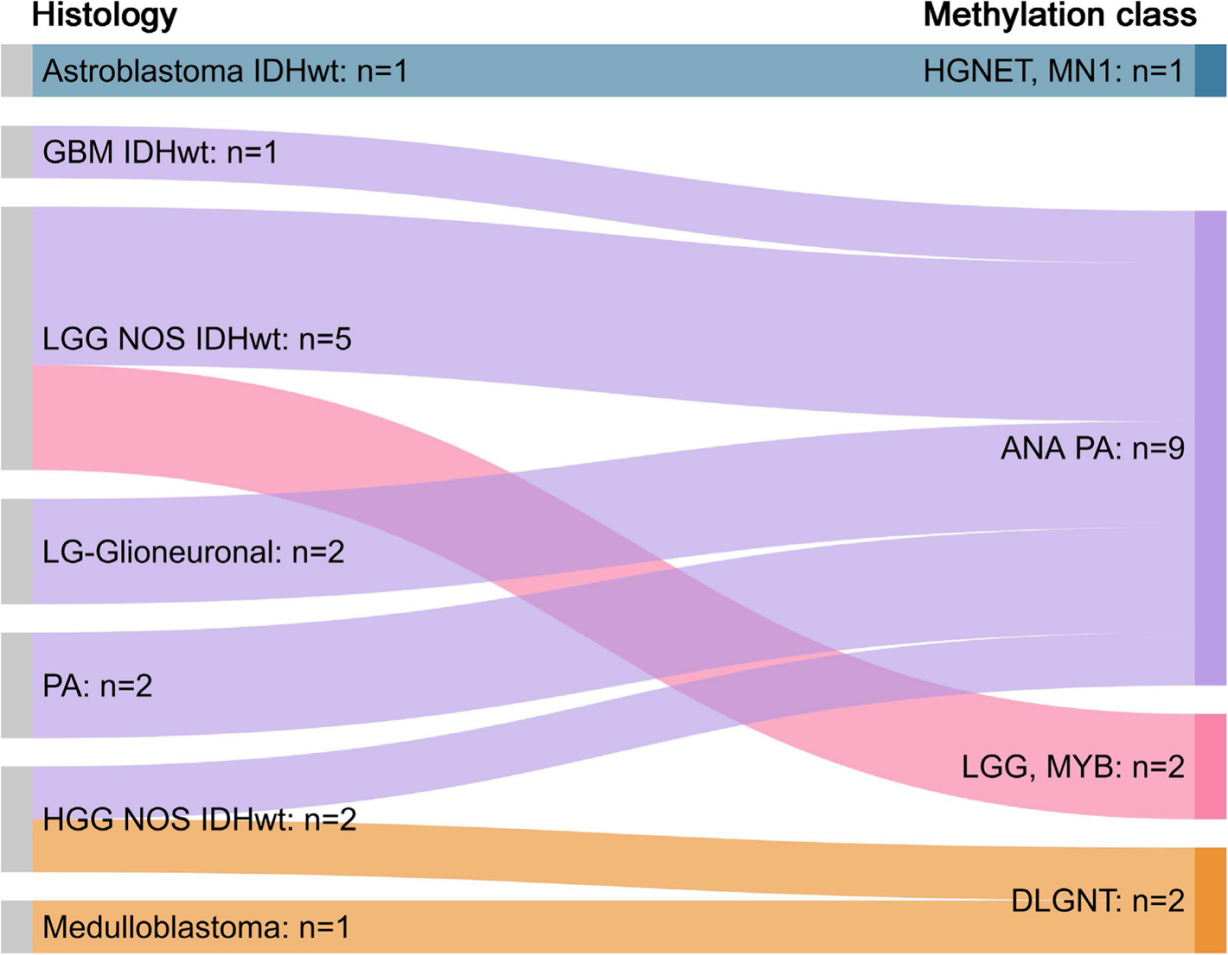
CNS tumours of varied histology resolving into new entities defined by their methylation profile. IDHwt: IDH-wildtype; GBM IDHwt: Glioblastoma, IDH-wildtype; LGG NOS IDHwt: Low-grade glioma not otherwise specified, IDH-wildtype; LG-Glioneuronal: Low-grade glioneuronal tumour; PA: Pilocytic astrocytoma; HGG NOS IDHwt: High-grade glioma not otherwise specified, IDH-wildtype; HGNET, MN1: CNS high-grade neuroepithelial tumour with MN1 alteration; ANA PA: Anaplastic pilocytic astrocytoma (anaplastic astrocytoma with piloid features); LGG, MYB: Low-grade glioma with MYB alteration; DLGNT: diffuse leptomeningeal glioneuronal tumour

In our cohort of 179 brain tumour cases with a calibrated score of > 0.84 from adolescents and adults (16 years and older) we have identified the following new biological entities defined by methylation classes: CNS high-grade neuroepithelial tumour with MN1 alteration (HGNET, MN1) (*n* = 1, aged 19), [40], anaplastic astrocytoma with piloid features (ANA PA) (*n* = 9, aged 25–73) [28], diffuse leptomeningeal glioneuronal tumour (DLGNT) (*n* = 2, aged 38 and 46) [8] and low-grade glioma with MYB alteration (LGG, MYB) (*n* = 2, aged 17 and 33) (Fig. 5). In keeping with previous reports, these tumours had either non-specific low-grade or high-grade astrocytic morphology, primitive small cell histology, or appearances of histologically defined entities of astroblastoma and pilocytic astrocytoma [8, 27, 28].

These new molecular entities were originally identified by DNA-methylation profiling and since then have been found to have characteristic recurrent genetic alterations. Therefore, whilst possible to diagnose them with conventional molecular methods involving DNA or RNA sequencing, in routine practice, in our opinion, the DNA methylation-based diagnostic approach is efficient and cost-effective for their identification.

#### IDH-wildtype gliomas with low-grade morphology

**Fig. 6:**
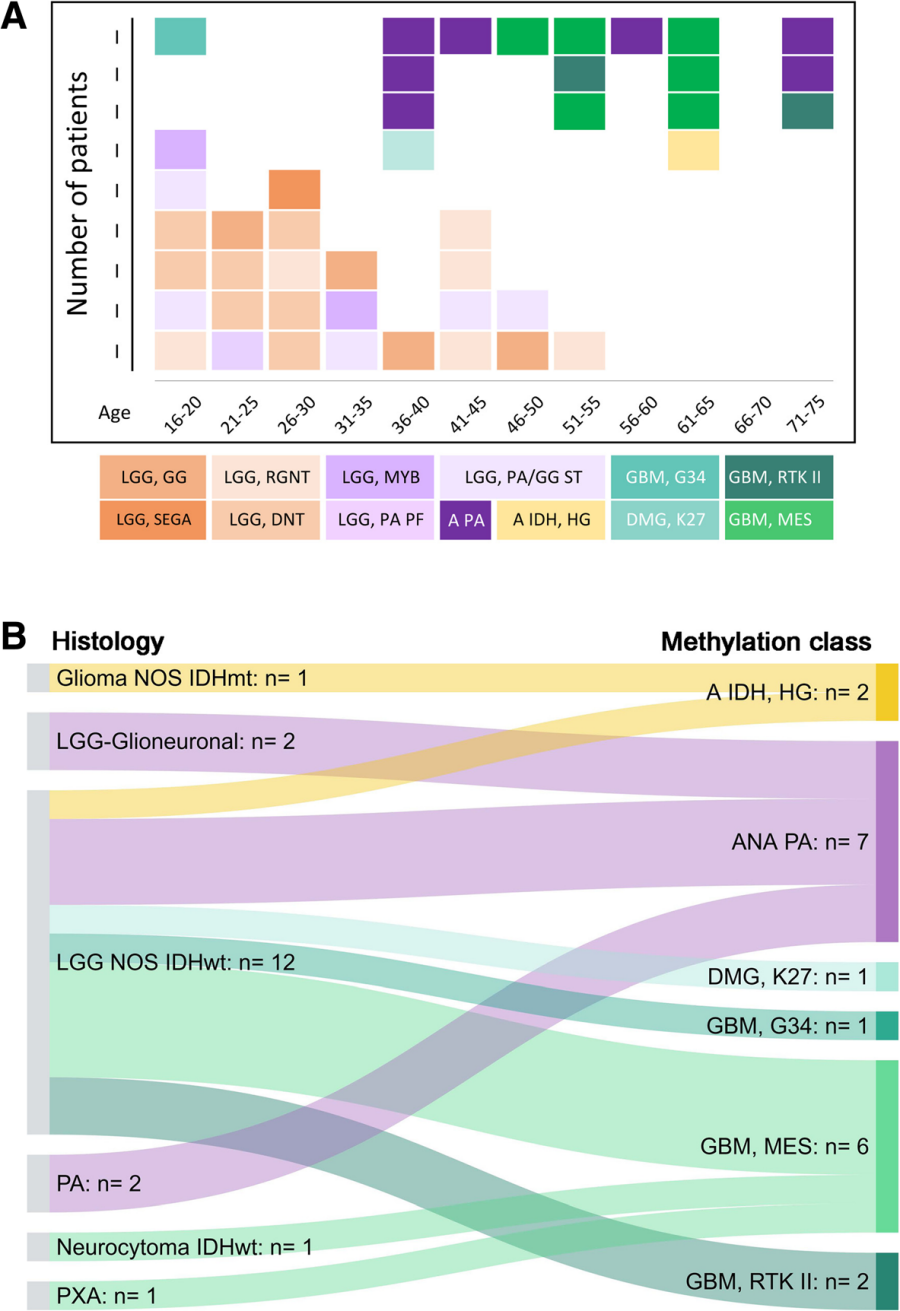
**a**, the outcome of methylation profiling of 44 IDH-wildtype CNS tumours with low-grade histology. Of these, 26 resolved into methylation classes associated with low-grade behaviour, and 18 resolved into entities associated with high-grade behaviour. **b**, shows how various tumours manifesting with low-grade histology resolve into distinct high-grade methylation classes. *Abbreviations* in **a**: LGG, GG: Low-grade glioma, ganglioglioma; LGG, SEGA: Low-grade glioma, subependymal giant cell astrocytoma; LGG, RGNT: Low-grade glioma, rosette forming glioneuronal tumour; LGG, DNT: Low-grade glioma, dysembryoplastic neuroepithelial tumour; LGG, MYB: Low-grade glioma with MYB alteration; LGG, PA PF: Low-grade glioma, pilocytic astrocytoma in posterior fossa; LGG, PA/GG ST: Low-grade glioma, pilocytic astrocytoma ganglioglioma spectrum in supratentorial compartment; A PA: Anaplastic pilocytic astrocytoma (anaplastic astrocytoma with piloid features); A IDH, HG: IDH-mutant high-grade astrocytoma; GBM, G34: H3 G34-mutant glioblastoma; DMG, K27: H3 K27-mutant diffuse midline glioma; GBM, RTK II: IDH-wildtype glioblastoma, RTK II subclass; GBM, MES: IDH-wildtype glioblastoma, mesenchymal subclass. *Abbreviations in* **b**: Glioma NOS IDHmt: IDH-mutant glioma, not otherwise specified; LGG-Glioneuronal: Low-grade glioma or glioneuronal tumour; LGG NOS IDHwt: IDH-wildtype low-grade glioma not otherwise specified; PA: Pilocytic astrocytoma; Neurocytoma, IDHwt: IDH-wildtype neurocytoma; PXA: pleomorphic xanthoastrocytoma; A IDH, HG: IDH-mutant high-grade astrocytoma; ANA PA: Anaplastic pilocytic astrocytoma (anaplastic astrocytoma with piloid features); DMG, K27: H3 K27-mutant diffuse midline glioma; GBM, G34: H3 G34-mutant glioblastoma; GBM, MES: IDH-wildtype glioblastoma, mesenchymal subclass; GBM, RTK II: IDH-wildtype glioblastoma, RTK II subclass

Forty-four tumours with glial or glioneuronal morphology, without histological high-grade features (such as hypercellularity, brisk mitotic activity, microvascular proliferation and/or necrosis) and without mutations in the *IDH1* (R132) or *IDH2* (R172) *or BRAF* (V600) genes were analysed with the Classifier and had a calibrated score of 0.84 and higher. For 18 (41%) of these tumours, the Classifier returned the diagnosis of IDH-wildtype glioblastoma, IDH-mutant high-grade astrocytoma, anaplastic pilocytic astrocytoma or H3 K27M- or G34R/V-mutant glioma (the discrepant IDH-mutant case was confirmed to carry an *IDH* mutation on repeat sequencing). The remaining 26 (59%) were classified as various low-grade glial or glioneuronal tumour entities (Fig. 6). The average age for tumours classified as low-grade tumour entities was 30.8 years and for those classified as high-grade tumour entities 53.6 years (Fig. 6).

#### IDH-wildtype gliomas with high-grade morphology

**Fig. 7:**
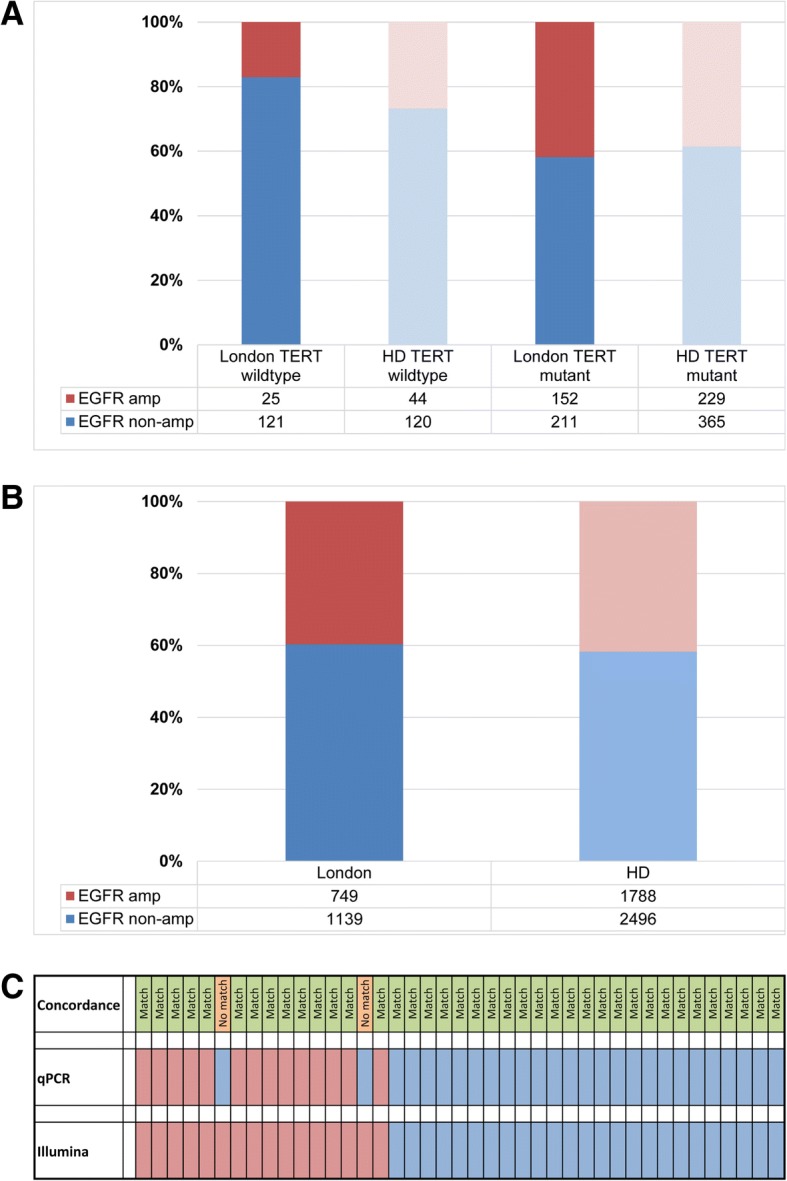
*EGFR* amplification in IDH-wildtype glioblastoma: **a**, comparison of our dataset with a previously published dataset [39] shows that the ratio of *EGFR* amplified and non-amplified, *TERT*-mutant GBM is similar to the published cohort (*p* = 0.3). Instead, the ratio of *EGFR* amplified and non-amplified, *TERT*-wildtype GBM is different between both cohorts (*p* = 0.04). **b**, comparison of the prevalence of *EGFR* status in GBM in our cohort (London, RT-PCR quantification) with those from the published dataset (“HD”, determined with the copy number readout from the methylation arrays) [39], shows no statistically significant difference (χ^2^ 2.3, *p* = 0.13). **c**, comparison of *EGFR* status in our cohort determined with Illumina arrays and with RT-PCR. There is a 95% concordance between both methods (χ^2^ 0.21, *p* = 0.64). *EGFR* was determined as amplified by RT-PCR where 6 and more copies were calculated with the CopyCaller™ software. *EGFR* data extracted from the copy number variation plot (downloadable from www.molecularneuropathology.org) were called amplified if the intensity was higher than 0.6 on a log2-scale [39]

The majority of IDH-wildtype high-grade gliomas are glioblastomas. Characteristic signatures are chromosome 7 gains and 10 losses, *EGFR* amplification and *TERT* promoter mutation [39]. In the cohort of [39] 1788 out of 4284 GBM (41.7%) were *EGFR* amplified (methylation classes: GBM_MYCN, GBM_RTK_I, GBM_RTK_II, GBM_RTK_III, GBM_MES, GBM_MID). In our cohort (2010–2018), we found 749 out of 1888 (39.7%) *EGFR* amplified GBM (6 and more copies), suggesting a nearly identical prevalence. From September 2015 till June 2018, 509 glioblastomas were tested for both *TERT* promoter mutations and *EGFR* status (Fig. 7). 121 were *TERT*-wildtype and *EGFR* non-amplified, 211 were *TERT*-mutant and *EGFR* non-amplified, 25 were *TERT*-wildtype and *EGFR* amplified and 152 were *TERT*-mutant and *EGFR* amplified. These data are largely comparable with a published dataset [39] (Fig. 7). In 41 cases of IDH-wildtype glioblastoma, *EGFR* status was assessed with both, methylation arrays and RTPCR. Only two cases showed a discrepant result, where the Illumina-derived plot showed amplification and the RT-PCR result revealed a copy number corresponding to non-amplified status, indicating a 95% concordance between both methods (χ^2^ 0.21, *p* = 0.64), (Fig. 7).

**Fig. 8:**
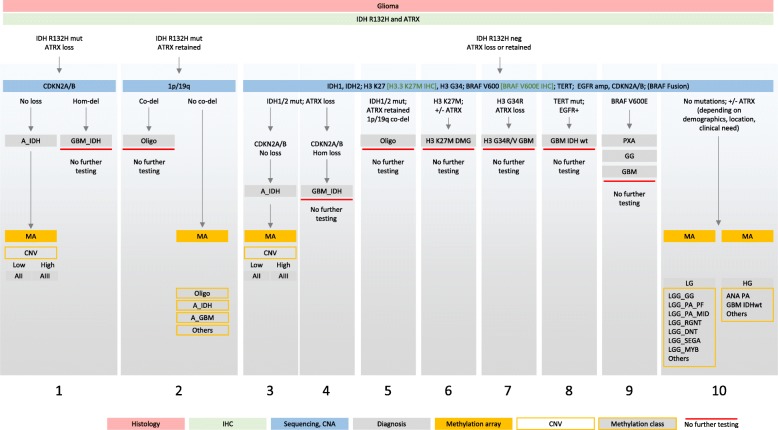
Diagnostic testing algorithm for gliomas in adults. The first layer is the histological assessment. The histological identification of a glial tumour is followed by the standard application of the antibodies IDH1 (R132H) and ATRX. This identifies a majority of IDH-mutant gliomas (column 1, 2). IDH-mutant astrocytomas with ATRX loss are further tested for *CDKN2A/B* homozygous deletion to stratify high risk from lower risk astrocytomas (column 1). Lower risk IDH-mutant astrocytomas are also assessed for copy number variation, a suggested prognostic factor. This is achieved by the readout of the copy number variation (CNV) component of the methylation arrays. IDH-mutant gliomas with retained ATRX expression (column 2) are further tested for 1p/19q co-deletion with a conventional copy number assay (in our practice combined with *TERT* promoter mutation analysis). Those IDH-mutant tumours which have retained ATRX expression and either no co-deletion or an ambiguous copy number result, are further tested with methylation array. This helps to differentiate IDH-mutant oligodendrogliomas from IDH-mutant astrocytomas or glioblastomas with retained ATRX protein expression. Gliomas which are negative for IDH1 R132H are further tested for a panel of biomarkers: *IDH1*, *IDH2*, H3 K27 and G34, *BRAF*, *TERT* promoter, *EGFR* and *CDKN2A/B*. IDH-mutant gliomas are shown in columns 3–5. The subsequent testing algorithm in column 3 is the same as in column 1. The outcomes from histone mutation testing are in columns 6, 7. A significant proportion of IDH-wildtype, EGFR-amplified and *TERT* promoter mutant glioblastomas are represented in column 8. These molecular entities do not require further testing at present. Also, the detection of a *BRAF* V600E mutation usually does not require further methylation array analysis (column 9). Those glial tumours with unequivocal histology (e.g. DNET, RGNT, ganglioglioma, IDH-wildtype GBM) are usually not further tested. Instead, those with non-characteristic and non-specific low-grade or high-grade histology and inconclusive molecular profile undergo methylation array analysis to inform of the methylation class which may also suggest candidate mutations that can be further tested for subsequent validation, such as rare mutations in histone variant encoding genes other than *H3F3A* (column 10). Often the methylation analysis also serves as a risk stratifier

A comprehensive table outlining a suggested diagnostic test algorithm, combining histology, immunohistochemical markers, conventional molecular tests and methylation arrays in low-grade and high-grade gliomas is shown in Fig. 8. We choose this approach as the consumable cost for Sanger sequencing and copy number assays are considerably below those of methylation arrays (approximately 10% of the cost) and the turnaround times allow for communication of test results within approximately 7 working days. Therefore, we choose methylation arrays as a first-line approach typically for small biopsies for which we predict an inconclusive outcome with our “conventional” molecular test portfolio, and for cases with an unusual histological presentation. In our routine practice, the first-line approach is usually as suggested in Fig. 8.

#### Ependymal tumours

**Fig. 9:**
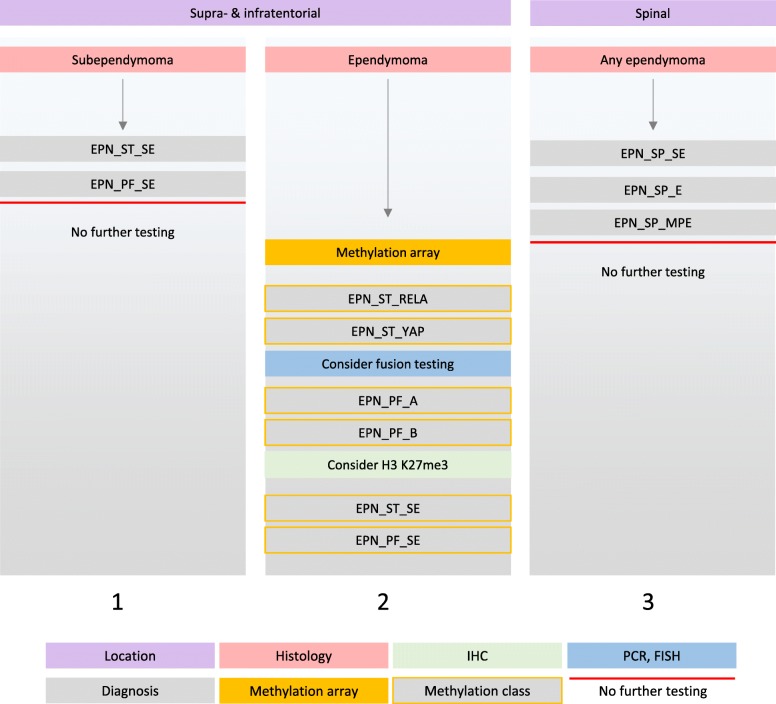
Diagnostic algorithm for ependymomas. In our diagnostic practice ependymomas in adults are infrequent. We first stratify the tumours by location and histological appearance. Identification of subependymomas is histologically straightforward and these tumours undergo no further testing (column 1). Supra- and infra-tentorial ependymomas are directly tested with methylation array (column 2). *RELA* and *YAP* fusions (and p65 and L1CAM IHC) may be further tested depending on the Classifier result. In our practice, EPN_PF_A are practically non-existent in the adult population, but H3 K27me3 expression status is technically straightforward and affordable and can be tested with IHC for completeness. A small proportion of supratentorial ependymomas with “classical” histology may be reclassified as subependymoma. Spinal tumours (column 3) are clinically low risk and their outcome is mainly determined by the extent of the surgical removal. Unless there is a specific clinical need or unusual histology, spinal tumours are not further tested with methylation arrays. *Abbreviations*: EPN_ST_SE: supratentorial subependymoma; EPN_PF_SE: posterior fossa subependymoma; EPN_ST_RELA: supratentorial ependymoma with RELA fusion; EPN_ST_YAP: supratentorial ependymoma with YAP fusion; EPN_PF_A: posterior fossa ependymoma group A; EPN_PF_B: posterior fossa ependymoma group B; EPN_SP_SE: spinal subependymoma; EPN_SP_E: spinal ependymoma; EPN_SP_MPE: spinal myxopapillary ependymoma

**Fig. 10:**
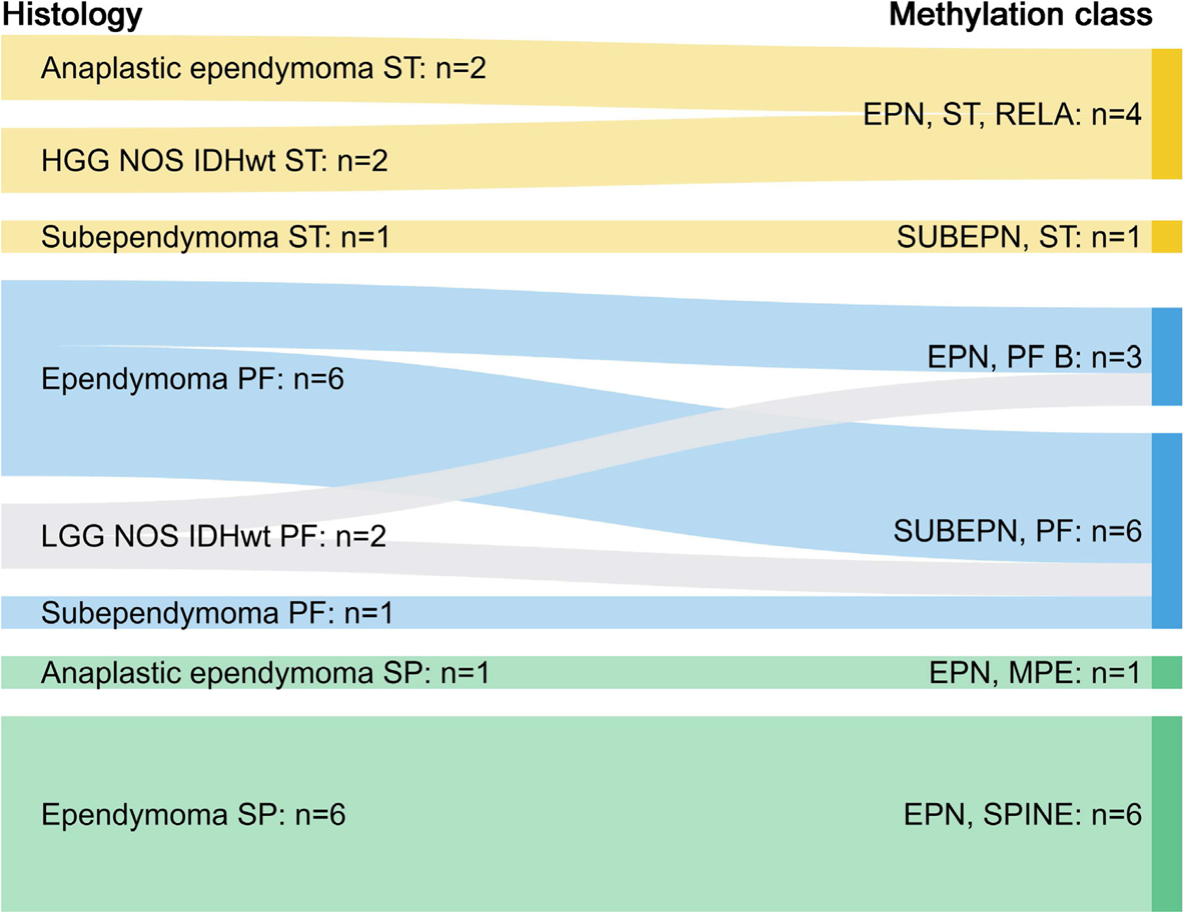
Refinement of diagnosis or identification of ependymomas through methylation profiling. Tumours with the histological diagnosis (left) of ependymoma or low-grade glioma, NOS were analysed for risk stratification or to establish a diagnosis. Only the tumours confirmed by the Classifier as ependymal are included in this diagram. *Abbreviations*: ST: supratentorial; PF: posterior fossa; SP: spinal; HGG NOS IDHwt ST: supratentorial high-grade glioma, not otherwise specified, IDH-wildtype; LGG NOS IDHwt PF: posterior fossa low-grade glioma, not otherwise specified, IDH-wildtype; EPN_ST, RELA: supratentorial ependymoma with RELA fusion (EPN_ST_RELA); SUBEPN, ST: supratentorial subependymoma (EPN_ST_SE); EPN, PF B: posterior fossa ependymoma group B (EPN_PF_B); SUBEPN, PF: posterior fossa subependymoma (EPN_PF_SE); EPN, MPE: spinal myxopapillary ependymoma (EPN_SP_MPE); EPN, SPINE: spinal ependymoma (EPN_SP_E)

Ependymal tumours located supratentorial or in the posterior fossa are submitted for the methylation analysis specifically for risk stratification purposes [24, 25] (Fig. 9). In our experience, the greatest discrepancy between the histological diagnosis and methylation class relates to the subependymoma entity in tumours located in the posterior fossa (Fig. 10). All but one ependymal tumour with the histology reported as classical ependymoma, WHO grade II, had a methylation profile of subependymoma. This finding is in line with earlier recommendations [24] that treatment decisions outside of clinical trials should not be based on a histologically assigned WHO grade. The Classifier also has helped in accurately diagnosing a subependymoma of which only small amounts of tissue were available, precluding a definitive histological diagnosis. An overview of the changes of diagnosis for ependymal tumours is given in Fig. 10. In another instance, the Classifier prompted us to change the diagnosis from histologically diagnosed anaplastic ependymoma to a *BRAF* V600E-mutant pleomorphic xanthoastrocytoma. This tumour underwent methylation array analysis specifically for the risk stratification after resection of a recurrence of a presumed ependymoma. The Classifier result (of anaplastic) PXA prompted us to test for, and confirm the *BRAF* V600E mutation, and the patient underwent treatment with BRAF inhibitors. Another tumour with histological features of anaplastic ependymoma was reclassified as H3 K27M-mutant diffuse midline glioma, and this mutation was confirmed subsequently by IHC and *H3F3A* gene sequencing.

## Conclusion

We report here a single centre experience of the implementation of methylation arrays into routine practice for algorithmic classification of brain tumours. In contrast to a paediatric setting, where a significant proportion of tumours undergoes methylation-based classification for risk stratification (such as ependymoma, medulloblastoma), methylation profiling for this purpose constitutes only a minority of our analysis of adult brain tumours. The combination of histological assessment with conventional molecular testing (i.e. IHC, targeted sequencing, copy number assay) is in our practice the first line diagnostic approach and is adequate for the majority of intrinsic tumours, such as IDH-mutant astrocytomas, oligodendrogliomas, histone-mutant, or *EGFR*-amplified, *TERT* promoter-mutant IDH-wildtype glioblastomas. By far the most common reason for using the methylation-based classification in adult practice is the need to obtain a more accurate and clinically relevant diagnosis for tumours with unusual, non-specific or non-representative histology and where the molecular testing does not yield diagnostically informative results. We found a change of diagnosis in approximately 25% of patients, refinement in approximately 50% and confirmation of the diagnosis in 25%. In a proportion of cases where the diagnosis changed, there was a significant impact on treatment and clinical management, and in others, the provision of accurate integrated diagnosis prevented from unnecessary, potentially harmful treatment. Our cohort further highlights the essential role of the methylation array as a diagnostic tool in advanced brain tumour diagnostics, when integrated into the diagnostic pathway in a structured fashion as outlined in Fig. 8 and Fig. 9.
